# Maternal vaccine hesitancy towards COVID-19 immunisation of children in Qatar: a population-based cross-sectional study

**DOI:** 10.4178/epih.e2022056

**Published:** 2022-07-06

**Authors:** Shuja Reagu, Suruchi Mohan, Johnny Awwad, Majid Alabdulla

**Affiliations:** 1 Department of Psychiatry, Mental Health Services, Hamad Medical Corporation, Doha, Qatar; 2 Weill Cornell Medicine, Doha, Qatar; 3 Department of Obstetrics and Gynaecology, Sidra Medicine, Doha, Qatar

**Keywords:** COVID-19, Vaccination hesitancy, Vaccination refusal, Qatar

## Abstract

**OBJECTIVES:**

This study was conducted in Qatar to explore beliefs and attitudes among mothers towards coronavirus disease 2019 (COVID-19) vaccination for their children and to understand major factors influencing vaccine hesitancy among these mothers.

**METHODS:**

A population-based, online cross-sectional survey was conducted between 15 October and 15 November 2020. A composite questionnaire incorporating a validated vaccine hesitancy tool was developed and administered in both English and Arabic. Approval was obtained from the local ethics committee. Participation was voluntary and offered to all adult residents of Qatar through an online link available on social media platforms and local news portals. Only adult respondents who self-identified as mothers were included in the present study. No personal identifying data were collected.

**RESULTS:**

Of the mothers surveyed, 29.4% exhibited COVID-19 vaccine hesitancy regarding their children. This exceeded these mothers’ rate of personal vaccine hesitancy (27.5%). Hesitancy rates varied significantly with ethnicity, with the highest among Qatari mothers (51.3%). Intention to vaccinate children did not differ significantly between mothers who accepted the vaccine for themselves and those who did not. Overall, the main reported concerns related to long-term vaccine safety. To a significant extent, mothers relied most on self-directed research on vaccine safety for decision-making.

**CONCLUSIONS:**

The rate of maternal COVID-19 vaccine hesitancy exceeded both those mothers’ rate of personal vaccine hesitancy and the hesitancy rate in the general population. The intention to vaccinate children was independent of maternal vaccination history. Factors influencing maternal vaccine hesitancy differ from those influencing personal hesitancy and require an informed public health response.

## INTRODUCTION

The coronavirus disease 2019 (COVID-19) pandemic has brought the world to its knees, and vaccines are crucial in delivering us from this health catastrophe. Pandemics can be controlled through herd immunity, and the safest way to attain herd immunity is through vaccination [1].

To achieve herd immunity, a large proportion of the population must be immunised. The populations of many countries have high proportions of children under 18 years old, meaning that children must be vaccinated to achieve the immunisation levels required for herd immunity [2]. Moreover, highly exposed and vulnerable groups can be targeted via immunisation, making vaccines a more effective approach for reducing viral circulation than naturally acquired infection [1]. The success of any immunisation-based strategy depends on vaccine uptake. Delays in vaccine acceptance along with vaccine refusal or “vaccine hesitancy” [3] have been highlighted among the top 10 threats to global health by the World Health Organisation (WHO) [4]. Public perceptions that new COVID-19 vaccines were “rushed” or inadequately tested, as well as mistrust of new technology, have resulted in emerging reports of high levels of COVID-19 vaccine hesitation among many populations [5]. In particular, relatively low rates of COVID-19 vaccine acceptance have been noted in the Middle East and North Africa [6]; in Qatar, prior to the implementation of public vaccination programs, one-fifth of the population was reported as being hesitant to accept COVID-19 immunisation [7].

While children are susceptible to COVID-19 infection, most remain asymptomatic or mildly symptomatic [8]. However, as the pandemic has unfolded, attention has shifted to children as transmitters of the infection and, more recently, to concerns regarding children’s vulnerability to emerging COVID-19 variants. This has provided an impetus for COVID-19 immunisation drives directed toward children. Indeed, some nations have already approved the administration of COVID-19 vaccines to adolescents, and in Qatar, all children and young adults at or above the age of 12 years are being offered COVID-19 immunisation.

When planning large-scale immunisation of children, parental vaccine hesitancy must be considered. Trials of the vaccine among children have been disorganised and slow to enroll [9], and the resulting lack of data may fuel concerns and vaccine hesitancy among parents. Even prior to the onset of the COVID-19 pandemic, vaccine hesitancy among parents and caregivers towards routine childhood immunisations and influenza vaccination was a growing concern [10]. Worryingly, this hesitancy has contributed to recent outbreaks of vaccine-preventable illnesses, such as measles [11]. In Qatar, up to around one-third of parents (29%) have been reported to exhibit hesitancy toward routine childhood immunisation [12]. Abundant evidence exists that maternal knowledge, attitudes, and beliefs around vaccination influence childhood vaccination rates [13,14]. The influence of mothers on the vaccination of adolescent children against human papillomavirus has also been studied; in 2014, Berenson et al. [15] reported that mothers make 93% of decisions regarding human papillomavirus immunisation for adolescents. Researchers have suggested that in the context of immunisation—an emotion-laden situation requiring decisions that affect the welfare of children—mothers are particularly receptive to the risks of harm depicted in anti-vaccine media portrayals [16].

Even as data on caregiver hesitancy toward COVID-19 vaccination emerge [17,18], a gap exists in the understanding of maternal attitudes toward COVID-19 immunisation of their children. Clearly, maternal vaccine hesitancy must be explored, and effective forms of motivation for parents to accept immunisation must be determined. The results can help direct health education, with a view toward ensuring the success of public immunisation drives.

## MATERIALS AND METHODS

### Population studied

Participation was offered on a voluntary basis to all residents of Qatar through an online link to the survey via Hamad Medical Corporation (HMC) social media platforms. The survey was available in English and Arabic. Videos in both languages explaining the nature and rationale of the survey accompanied the invitation link.

For this study, respondents who identified themselves as mothers (women who answered in the affirmative regarding whether they had children) were included and their responses collected.

### Research materials

The survey comprised a composite questionnaire that included the Vaccination Attitudes Examination (VAX) tool, a validated vaccine hesitancy measurement instrument [19]. The VAX scale was created as a measure of general vaccination attitudes and, unlike other vaccine hesitancy scales, is not focused on a particular population or a specific vaccine. The questions in the VAX scale were designed and validated to measure 4 attitudes of vaccine hesitancy: mistrust of the benefit of vaccines, concerns about the effects and long-term safety of vaccines, suspicions around commercial profiteering through vaccines, and trust in natural immunity as superior to vaccine-induced immunity. These 4 attitudes have been shown to influence vaccine-related intentions [19]. The VAX tool presents statements designed to capture these attitudes to respondents, who then indicate the extent to which they agree or disagree with each statement. With the VAX scale, the survey could be used to explore participants’ attitudes toward COVID-19 immunisation, examine vaccine-related concerns, and collect relevant background data. The questionnaire design was guided by the WHO Strategic Advisory Group of Experts on Immunisation model of the assessment of vaccine hesitancy with regard to cultural/contextual factors, external influences, and vaccine-specific concerns [20]. The questionnaire was designed in English and translated into Arabic; then, the Arabic version was validated per published guidelines [21].

### Outcome measures

The outcome measures included: (1) Intention to vaccinate children: accept vaccination or admit vaccine hesitancy and (2) Factors influencing intention to vaccinate: Socio-demographic factors (including education, ethnicity, and cultural factors); Internal and external influences (including previous vaccination choices, general beliefs around immunisation, access to information, endorsement by health authorities, and trust in pharmaceutical companies as well as health systems); Vaccine-specific factors (including knowledge of and concerns around COVID-19 vaccination and perception of health risks from the vaccine).

### Statistical analysis

The online survey response data was collected and imported to Microsoft Excel (Microsoft Co., Redmond, WA, USA). The data were analysed using SPSS version 27.0 (IBM Corp., Armonk, NY, USA). Descriptive statistics were calculated with non-parametric analysis of continuous variables and chi-square analysis of discrete variables.

### Ethics statement

A population-based, cross-sectional online survey on attitudes to COVID-19 immunisation was conducted from 15 October to 15 November 2020. This study was part of that survey. Ethical approval was granted by the Medical Research Council (MRC) of the HMC (MRC approval 01-20-930). The HMC is the state health service that provides government-subsidized care to the nation.

## RESULTS

A total of 7,882 people participated in the survey on COVID-19 vaccine hesitancy, of whom 6,882 parents (2,882 women and 4,000 men) responded to questions about vaccination of their children. The results from the 2,882 mothers are presented here.

### Socio-demographic background of respondents

Table 1 details the socio-demographic backgrounds of the mothers. Arabic women constituted the largest proportion (41.4%), with Qatari nationals comprising 16.7%. Overall, the respondents were highly educated, with 79.8% reporting university-level education, and a majority were employed (70.3%; 65.7% salaried and 4.6% self-employed). The largest proportion of women was between 26 years and 35 years old (34.9%), followed closely by those between 36 years and 45 years old (34.7%). The vast majority of the group had completed their childhood vaccinations (94.6%). When asked about influenza vaccination over the prior 3 years, of the participants who responded (53.2%), only 4 had not been immunised at all against influenza (Table 1).

### Intention to vaccinate children against COVID-19

The women were first asked whether they intended to accept the COVID-19 vaccine for themselves. Of the respondents, 1,387 (48.1%) said they definitely or probably would accept the vaccine, while 792 (27.5%) exhibited vaccine hesitancy by responding that they definitely or probably would not accept the vaccine (Table 2). When asked whether they would accept COVID-19 vaccination for their children, 1,358 (47.1%) responded that they definitely or probably would do so, while 847 (29.4%) were vaccine-hesitant regarding their children.

Among the 1,387 participants who would accept the vaccine for themselves, a majority (1,248 mothers, 90.0%) were accepting of COVID-19 immunisation for their children, with only 15 mothers (1.1%) saying that they would hesitate in accepting COVID-19 immunisation for their children. In contrast, the rate of maternal vaccine hesitancy among “self-hesitators” was 91.7%, differing significantly from the self-acceptor group (p<0.01). Of those who were undecided regarding personal vaccine acceptance, approximately one-fifth (99 mothers, 19.9%) responded that they would accept immunisation for their children (Table 2).

### Influencing factors

Further differentiating by ethnicity and nationality, Qatari nationals exhibited significantly higher levels of hesitancy towards COVID-19 immunisation for their children (at 51.3%) than other Arab nationals (39.1%), Asians (10.8%) and mothers of other nationalities (30.9%) (chi-square test, 174.23; p<0.01). Table 3 shows the results regarding maternal vaccine hesitancy by participant ethnicity and nationality.

Participants were asked which external endorsements could impact their decisions around accepting COVID-19 vaccination, and Table 4 shows the responses. Overall, the most commonly cited influence was the reading of research about vaccine safety and effectiveness. The other commonly cited factors were endorsement of the vaccine by the respondent’s physician or a health service and endorsement by the WHO. Endorsement of immunisation by a public figure was the least frequently cited influence. However, when the responses were stratified by intention regarding COVID-19 vaccination of children (i.e., acceptors vs. hesitators), marked differences in influencing factors were noted. Vaccine acceptors were significantly more likely to be influenced by external endorsements (i.e., endorsements by the WHO, health ministry, public figure or health service/professional), at 61.6%, compared to vaccine hesitators at 14.3% (*χ*^2^-test, 545.60; p<0.01). Vaccine hesitators were significantly more likely than acceptors to rely on reading scientific research on vaccine efficacy (*χ*^2^-test, 58.93; p<0.01).

When asked to specify (via free text) other influences that may impact decisions around accepting immunisation, the 2 recurrent themes among hesitators were that “nothing else would add to my vaccine confidence” or that “more years of research data on the vaccine” were needed. Among the vaccine acceptors, the most commonly cited “other” influence was endorsement by bodies not listed in the choices.

### Attitudes, knowledge, and beliefs around COVID-19 vaccination

**Figure 1.**
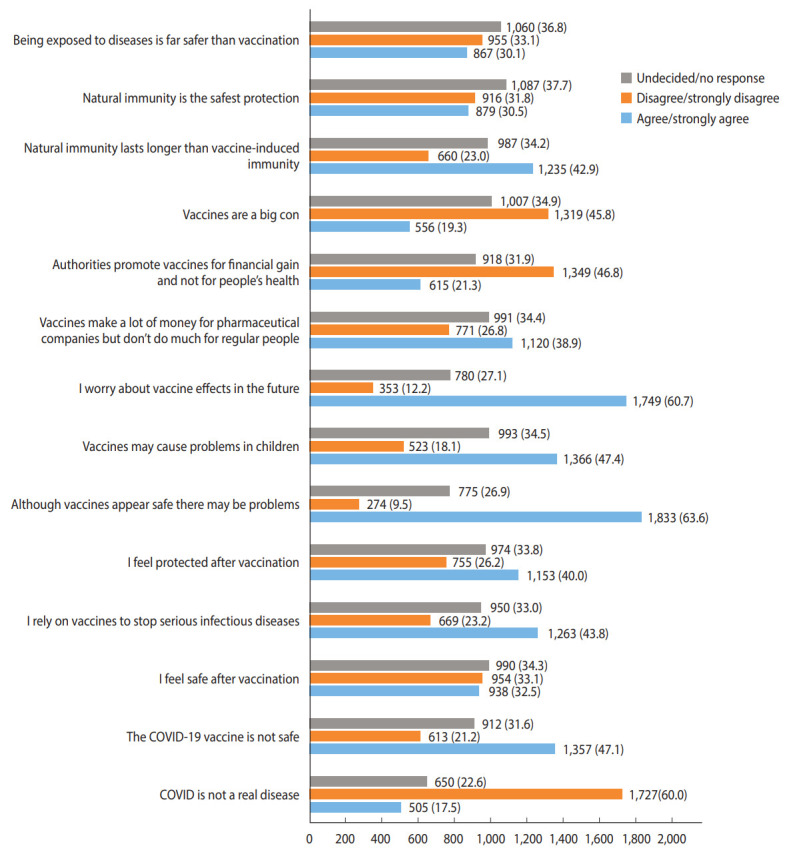
Attitudes, knowledge, and beliefs around coronavirus disease 2019 (COVID-19) vaccination (per the Vaccination Attitudes Examination tool). Values are presented as number (%).

The responses of the participants (n=2,882) to questions exploring attitudes, knowledge, and beliefs around COVID-19 vaccination, as measured with the VAX instrument, are shown in Figure 1. Of the respondents, 60.0% (1,727 mothers) acknowledged that COVID-19 was a real disease, but nearly half (47.1%, 1,357 mothers) perceived the vaccine for the disease as unsafe. Less than half of the group (43.8%, 1,263 mothers) reported generally relying on vaccination for protection against serious infections; 40.0% (1,153 mothers) reported feeling protected after vaccination, and 32.5% (938 mothers) said they felt safe after vaccination. Over half of the respondents (63.6%, 1,833 mothers) held the view that vaccines may appear safe but could be associated with future problems, and 60.7% (1,749 mothers) admitted to worrying about these potential future effects. Concerns that vaccines could cause problems in children were expressed by 47.4% of participants (1,366 mothers). A total of 38.9% of participants (1,120 mothers) perceived vaccines as money-makers for pharmaceutical companies, and approximately one-fifth (21.3%, 615 mothers) felt that authorities promoted vaccination for financial gain. Of the respondents, 19.3% (556 mothers) were overtly dubious of vaccines, considering them a “big con.” In total, 42.9% of the respondents (1,235 mothers) expressed the belief that natural immunity lasted longer than vaccine-induced immunity, and 30.5% (879 mothers) felt that natural immunity was the safest form of protection from infection. Notably, nearly one-third of respondents (30.1%, 867 mothers) felt that being exposed to infection was safer than vaccination.

The responses of the mothers to the VAX items were analysed further to compare hesitators toward children’s immunisation with acceptors. Hesitators significantly more frequently expressed concerns that the COVID-19 vaccine had not been fully tested (*χ*^2^-test, 226.70; p<0.01), concerns that vaccines may be associated with future problems (*χ*^2^-test, 25.63; p<0.01), perceptions that vaccines may have unforeseen effects on children (*χ*^2^-test, 117.43; p<0.01), worries about unknown future effects of vaccines (*χ*^2^-test, 69.97; p<0.01), perceptions that vaccines were money-makers for pharmaceutical companies (*χ*^2^-test, 143.73; p<0.01), views that authorities promoted immunisation for financial gain (*χ*^2^-test, 181.89; p < 0.01) and perceptions of vaccines as a “con” (*χ*^2^-test, 82.35; p<0.01). maternal vaccine hesitators were significantly more likely than acceptors to believe that natural immunity lasted longer than immunisation (*χ*^2^-test, 55.67; p<0.01), that natural exposure provided the safest protection (*χ*^2^-test, 97.27; p<0.01) and that natural exposure was safer for the immune system than vaccination (*χ*^2^-test, 141.21; p<0.01).

## DISCUSSION

The main finding of this study is a high frequency of hesitancy (29.4%) among mothers toward COVID-19 vaccination of their children. This figure was even higher, at 51.3%, among mothers who were Qatari nationals. These hesitancy rates were also higher than the reported rate of 20% for the nationwide vaccine hesitancy population survey (n=7,821) in Qatar [7], as well as the 25% hesitancy rate among perinatal women in Qatar [22] towards COVID-19 vaccination for their children. Data on COVID-19 vaccine hesitancy are still emerging, and rates of personal vaccine hesitancy have been reported at 27-28% in studies across multiple nations [23,24]. In a recent study, determinants of parental hesitancy towards COVID-19 immunisation in a Chinese city were explored. Survey data from 1,788 parents revealed a parental vaccine hesitancy rate of 52.5%, with hesitancy significantly more common among mothers than fathers (57.5 vs. 41.7%) [25]. A survey of willingness to vaccinate children against COVID-19 among caregivers attending emergency departments with their children across 6 countries reported that 65% (n=1,005) of caregivers said they would accept COVID-19 vaccination for their children, with less hesitancy among fathers than mothers [17]. The higher vaccine hesitancy rates among mothers are mirrored by the results of the present study and are unsurprising, as the literature abounds with evidence for higher maternal than paternal hesitancy levels for traditional childhood immunisation [12-14]. The fact that mothers are important decision-makers in accepting vaccinations for children and adolescents has been thoroughly documented [13-15], increasing the importance of these findings. Mothers should therefore receive attention from healthcare planning authorities for targeted health education interventions.

In the context of adolescent immunisation, mothers have been shown to be more susceptible to media reports of adverse effects and risks, potentially due to prioritization of “loss aversion” in the emotion-laden decision-making process [16,26]. Additionally, online misinformation has been shown to affect public sentiment and directly influence COVID-19 vaccine hesitancy [27]. This is significant considering our finding that overall, mothers relied most on their own reading about the safety and efficacy of the vaccine when making decisions around vaccination. This finding was particularly pronounced among the vaccine hesitators. It is therefore imperative that the health authorities focus on providing access to reliable vaccine information through preferred media platforms, congruent with the current digital world, while tackling rampant online vaccine misinformation.

The mothers in this study exhibited high levels of education, childhood immunisation, and influenza vaccination, all of which are factors associated with greater vaccine acceptance [18,25]. However, they still demonstrated hesitancy toward COVID-19 immunisation for their children. Clearly, therefore, distinct concerns factor into parental decision-making regarding the COVID-19 vaccine. The main concerns expressed by this group in relation to COVID-19 vaccination of their children revolved around vaccine safety and the risk of future health problems due to immunisation. Indeed, COVID-19 vaccine-specific concerns have been cited as primary in relation to the acceptance of the vaccine for children [17,18,25]. In this study, the hesitators toward children’s vaccination most commonly cited “more long-term safety data” as the factor that would influence their decision to accept vaccination of their children. Notably, as this is a novel vaccine, long-term safety data may take years to collect. However, the situation has been compounded by the slow start and lack of a systematic approach to COVID-19 vaccine trials on children [9]. Some researchers have emphatically opposed the default position of delaying the initiation of phase 2 trials in children while awaiting the results of efficacy trials in adults [28]. Indeed, at the time this survey was conducted in October-November 2020, only Pfizer had announced the enrolment of 12-year-olds to 15-year-olds in trials for its COVID-19 vaccine, with Moderna trials beginning in December 2020 and the Oxford group starting trials in children in February 2021 [29]. The relative lack of vaccine trial data for children can fuel parental anxiety and potentially derail COVID-19 immunisation drives directed at children, and may have impacted participant attitudes at the time of this survey. Therefore, governments should prioritise the collection and dissemination of evidence regarding the safety of this vaccine for children [30].

A concerning sentiment of public mistrust and apprehension exists around the COVID-19 vaccine [27]. In this study, hesitators were significantly more likely than acceptors to be dubious of the vaccine, regarding it a money-making “con” and mistrusting the intentions of pharmaceutical companies and governments promoting immunisation. Governments must assuage these concerns and garner public trust through transparent, honest, factual and targeted public health education measures. Additionally, this may be a cue for stakeholders to turn to COVID-19 vaccination education strategies that take the “decisional balance” approach [31], weighing the risk of contracting infection against the potential risks of the vaccine.

The hesitancy rate regarding children differed significantly between mothers who accepted and those who were hesitant toward COVID-19 immunisation for themselves, with acceptors showing less hesitancy about vaccinating their children. Thus, general attitudes towards vaccination must be addressed in promoting vaccination among mothers for themselves and for their children. Altruistic themes intended to motivate COVID-19 immunisation have formed prominent parts of public health campaign messages in many countries, including Qatar [32,33]. While part of the strategy behind immunising children is to target these potential spreaders of COVID infection, an altruistic theme in promoting vaccination for children may be effective.

Finally, the results of this study must be interpreted in the cultural context of Qatar. Qatar is a high-income nation with high overall educational attainment and employment levels [34]; therefore, it is comparable to developed Western nations, and research cited from these regions is applicable. It is also a multicultural, majority-migrant state [34]. The present study revealed a marked ethnicity-based disparity in parental hesitancy rates towards COVID-19 vaccination, with Qatari mothers being the most hesitant, followed by mothers of non-Qatari Arabic origin. Cultural factors (along with vaccine safety) are the most important factors influencing acceptance of immunisation [35]. Therefore, exploring cultural influences on vaccine-related decision-making is critical when planning immunisation programs for this region.

The strength of this study is its inclusion of results from a population-based survey with many participants, conducted at a time when trials of COVID-19 vaccines among children had just commenced. In this manner, it allows insight into parental attitudes towards the prospect of vaccination of their children. These insights could help plan the communication and organization of effective immunisation drives for children and adolescents. Furthermore, a validated vaccine hesitancy tool was used, and the set outcome measures were based on internationally accepted standards regarding vaccine hesitancy. The study was limited in that it was conducted at a time when no data on vaccine safety among children were available, and the immunisation of children had not yet begun in Qatar. A few months after the survey was conducted, Qatar started COVID-19 immunisation for children at least 12 years old. Therefore, attitudes and perceptions may evolve with time due to greater availability of vaccine safety data, additional experiences with the vaccination of adults, and preliminary experiences in vaccinating children. Finally, the survey was administered online and was available in only 2 languages, potentially limiting participation based on language and digital access barriers.

The results of this study reveal maternal hesitancy toward COVID-19 immunisation of their children that is independent of the mothers’ prior vaccine behaviours. Self-vaccine hesitators also appeared to resist vaccinating their children against COVID-19 in this study. In this decision-making process, vaccine-specific safety concerns predominate among mothers and must be recognized and addressed via a tailored and targeted approach. Stakeholders in vaccine communication campaigns should be informed that efforts to counter anti-vaccine attitudes that have hitherto been heavily relied on for adult COVID-19 vaccination campaigns (such as providing scientific information, promoting public education, and capitalizing on altruistic motivations) should be continued to motivate vaccine-hesitant parents. Most importantly, we call for support of robust vaccine trials among children and adolescents to provide much-needed safety and efficacy data to assuage mothers’ concerns.

## Figures and Tables

**Table 1.** Socio-demographic characteristics and prior vaccination status of the study participants

Characteristics	n (%)	Response not recorded (n)
Ethnicity/nationality		
Arab (Qatari)	480 (16.7)	0 (0.0)
Arab (non-Qatari)	713 (24.7)	
Asian	993 (34.5)	
Other	696 (24.1)	
Level of education		
University	2,301 (79.8)	293 (10.2)
High school	223 (7.7)	
Vocational training	61 (2.1)	
Other	4 (0.1)	
Occupation		
Employed (salaried)	1,893 (65.7)	0 (0.0)
Employed (self)	134 (4.6)	
Unemployed	660 (22.9)	
Retired	195 (6.8)	
Age (yr)		
18-25	109 (3.8)	9 (0.3)
26-35	1,006 (34.9)	
36-45	999 (34.7)	
≥46	759 (26.3)	
Childhood immunisation		
Completed childhood vaccinations	2,727 (94.6)	
Did not complete childhood vaccinations	155 (5.4)	
No. of influenza vaccines in past 3 yr		
3	764 (26.5)	1,350 (46.8)
2	265 (9.2)	
1	503 (17.5)	
0	4 (0.1)	
Currently pregnant	86 (3.0)	
Currently breastfeeding	255 (8.8)	

**Table 2.** Intentions to vaccinate self and children

Variables		No. of vaccine acceptors who would definitely or probably accept the COVID-19 vaccine	Undecided on taking the COVID-19 vaccine	No. of vaccine hesitators who would probably not or definitely not accept the COVID-19 vaccine	No. of respondents who did not record a choice on intent to vaccinate against COVID-19
Vaccine intention among the entire group (n=2,882)					
	COVID-19 vaccination for self	1,387 (48.1)	498 (17.2)	792 (27.5)	205 (7.11)
	COVID-19 vaccination for children	1,358 (47.1)	677 (23.5)	847 (29.4)	0 (0.0)
Vaccine intention for their children among vaccine “self-acceptors” (n=1,387)					
	COVID-19 vaccination for children	1,248 (90.0)	124 (8.9)	15 (1.1)	-
Vaccine intention for their children among vaccine “self-hesitators” (n=792)					
	COVID-19 vaccination for children	11 (1.4)	55 (6.9)	726 (91.7)	-
Vaccine intention for their children among those “undecided” towards vaccine for self (n=498)					
	COVID-19 vaccination for children	99 (19.9)	293 (58.8)	106 (21.3)	-

Values are presented as number (%). COVID-19, coronavirus disease 2019.

**Table 3.** Vaccine hesitancy for children by participants’ ethnicity/nationality

Participant ethnicity/nationality	Vaccine hesitators for their children within each population subgroup (%)	p-value^1^
Qatari (n=480)	51.3	<0.01
Arab (non-Qatari) (n=713)	39.1	
Asian (n=993)	10.8	
Other (n=696)	30.9	

1 Chi-square test.

**Table 4.** Endorsements or influences that would increase confidence in accepting the vaccine^1^

Variables	Participants (n=2,882)	Responses by acceptors of children’s immunisation against COVID-19 (n=1,358)	Responses by hesitators toward children’s immunisation against COVID-19 (n=847)	Statistical significance	
				χ^2^-test	p-value
Endorsement by my doctor/health service	428 (14.9)	280 (20.6)	45 (5.3)	546.60	<0.01
Endorsement by a public figure	17 (0.6)	15 (1.1)	2 (0.2)		
Endorsement by the Ministry of Health	376 (13.0)	267 (19.7)	30 (3.5)		
Endorsement by the WHO	401 (13.9)	275 (20.2)	44 (5.2)		
Positive feedback from friends/family	179 (6.2)	68 (5.0)	51 (6.0)		0.30
Reading scientific research on its effectiveness	905 (31.4)	299 (22.0)	368 (43.4)	58.93	<0.01
Other (please specify)	312 (10.8)	30 (2.2)	239 (28.2)		

COVID-19, coronavirus disease 2019; WHO, World Health Organisation.

1 Participants could choose multiple endorsements/influences or none, as applicable; Of the 2,882 mothers, 1,358 were acceptors and 847 were hesitators towards children’s vaccination, while 677 were undecided, as shown in Table 2.

## References

[1] Fontanet A, Cauchemez S (2020). COVID-19 herd immunity: where are we?. Nat Rev Immunol.

[2] Klass P, Ratner AJ (2021). Vaccinating children against Covid-19 - the lessons of measles. N Engl J Med.

[3] MacDonald NE, SAGE Working Group on Vaccine Hesitancy (2015). Vaccine hesitancy: definition, scope and determinants. Vaccine.

[4] https://www.who.int/news-room/spotlight/ten-threats-to-global-health-in-2019.

[5] Dror AA, Eisenbach N, Taiber S, Morozov NG, Mizrachi M, Zigron A (2020). Vaccine hesitancy: the next challenge in the fight against COVID-19. Eur J Epidemiol.

[6] Sallam M (2021). COVID-19 vaccine hesitancy worldwide: a concise systematic review of vaccine acceptance rates. Vaccines (Basel).

[7] Alabdulla M, Reagu SM, Al-Khal A, Elzain M, Jones RM (2021). COVID-19 vaccine hesitancy and attitudes in Qatar: a national cross-sectional survey of a migrant-majority population. Influenza Other Respir Viruses.

[8] Kamidani S, Rostad CA, Anderson EJ (2021). COVID-19 vaccine development: a pediatric perspective. Curr Opin Pediatr.

[9] Mintz K, Jardas E, Shah S, Grady C, Danis M, Wendler D (2021). Enrolling minors in COVID-19 vaccine trials. Pediatrics.

[10] Sadaf A, Richards JL, Glanz J, Salmon DA, Omer SB (2013). A systematic review of interventions for reducing parental vaccine refusal and vaccine hesitancy. Vaccine.

[11] Phadke VK, Bednarczyk RA, Salmon DA, Omer SB (2016). Association between vaccine refusal and vaccine-preventable diseases in the United States: a review of measles and pertussis. JAMA.

[12] Novelli VM, Khalil N, Metarwah B, El-Baba F, Nahar R, Abu-Nahya M (1991). Childhood immunization in the State of Qatar: implications for improving coverage. Ann Saudi Med.

[13] Larson Williams A, Mitrovich R, Mwananyanda L, Gill C (2019). Maternal vaccine knowledge in low- and middle-income countriesand why it matters. Hum Vaccin Immunother.

[14] Danchin MH, Costa-Pinto J, Attwell K, Willaby H, Wiley K, Hoq M (2018). Vaccine decision-making begins in pregnancy: correlation between vaccine concerns, intentions and maternal vaccination with subsequent childhood vaccine uptake. Vaccine.

[15] Berenson AB, Laz TH, Hirth JM, McGrath CJ, Rahman M (2014). Effect of the decision-making process in the family on HPV vaccination rates among adolescents 9-17 years of age. Hum Vaccin Immunother.

[16] Argyris YA, Kim Y, Roscizewski A, Song W (2021). The mediating role of vaccine hesitancy between maternal engagement with antiand pro-vaccine social media posts and adolescent HPV-vaccine uptake rates in the US: the perspective of loss aversion in emotionladen decision circumstances. Soc Sci Med.

[17] Goldman RD, Yan TD, Seiler M, Parra Cotanda C, Brown JC, Klein EJ (2020). Caregiver willingness to vaccinate their children against COVID-19: cross sectional survey. Vaccine.

[18] Wang Q, Xiu S, Zhao S, Wang J, Han Y, Dong S (2021). Vaccine hesitancy: COVID-19 and influenza vaccine willingness among parents in Wuxi, China-a cross-sectional study. Vaccines (Basel).

[19] Martin LR, Petrie KJ (2017). Understanding the dimensions of anti-vaccination attitudes: the vaccination attitudes examination (VAX) scale. Ann Behav Med.

[20] https://www.who.int/groups/strategic-advisory-group-of-experts-on-immunization.

[21] Sousa VD, Rojjanasrirat W (2011). Translation, adaptation and validation of instruments or scales for use in cross-cultural health care research: a clear and user-friendly guideline. J Eval Clin Pract.

[22] Farrell T, Reagu S, Mohan S, Elmidany R, Qaddoura F, Ahmed EE (2020). The impact of the COVID-19 pandemic on the perinatal mental health of women. J Perinat Med.

[23] Boyon N COVID-19 vaccination intent is decreasing globally. https://www.ipsos.com/en/global-attitudes-covid-19-vaccine-october-2020.

[24] Lazarus JV, Ratzan S, Palayew A, Billari FC, Binagwaho A, Kimball S (2020). COVID-SCORE: a global survey to assess public perceptions of government responses to COVID-19 (COVID-SCORE-10). PLoS One.

[25] Zhang MX, Lin XQ, Chen Y, Tung TH, Zhu JS (2021). Determinants of parental hesitancy to vaccinate their children against COVID-19 in China. Expert Rev Vaccines.

[26] Motta M, Callaghan T, Sylvester S (2018). Knowing less but presuming more: Dunning-Kruger effects and the endorsement of anti-vaccine policy attitudes. Soc Sci Med.

[27] Loomba S, de Figueiredo A, Piatek SJ, de Graaf K, Larson HJ (2021). Measuring the impact of COVID-19 vaccine misinformation on vaccination intent in the UK and USA. Nat Hum Behav.

[28] Anderson EJ, Campbell JD, Creech CB, Frenck R, Kamidani S, Munoz FM (2021). Warp speed for coronavirus disease 2019 (COVID-19) vaccines: why are children stuck in neutral?. Clin Infect Dis.

[29] Mahase E (2021). Covid vaccine could be rolled out to children by autumn. BMJ.

[30] Opel DJ, Diekema DS, Ross LF (2021). Should we mandate a COVID-19 vaccine for children?. JAMA Pediatr.

[31] Williams L, Gallant AJ, Rasmussen S, Brown Nicholls LA, Cogan N, Deakin K (2020). Towards intervention development to increase the uptake of COVID-19 vaccination among those at high risk: outlining evidence-based and theoretically informed future intervention content. Br J Health Psychol.

[32] National Health Service England (2020). We must keep on protecting each other. https://www.hphigh.co.uk/attachments/download.asp?file=455&type=pdf.

[33] Centers for Disease Control and Prevention (2022). COVID-19: how to protect yourself and others. https://www.cdc.gov/coronavirus/2019-ncov/prevent-getting-sick/prevention.html.

[34] De Bel-Air F (2017). Demography, migration, and labour market in Qatar. https://gulfmigration.grc.net/media/pubs/exno/GLMM_EN_2017_03.pdf.

[35] Wilson RJ, Paterson P, Jarrett C, Larson HJ (2015). Understanding factors influencing vaccination acceptance during pregnancy globally: a literature review. Vaccine.

